# Age-specific transmission dynamic of mumps: A long-term large-scale modeling study in Jilin Province, China

**DOI:** 10.3389/fpubh.2022.968702

**Published:** 2022-11-07

**Authors:** Tianlong Yang, Yao Wang, Qinglong Zhao, Xiaohao Guo, Shanshan Yu, Zeyu Zhao, Bin Deng, Jiefeng Huang, Weikang Liu, Yanhua Su, Tianmu Chen

**Affiliations:** ^1^State Key Laboratory of Molecular Vaccinology and Molecular Diagnostics, School of Public Health, Xiamen University, Xiamen, China; ^2^Jilin Provincial Center for Disease Control and Prevention, Changchun, China

**Keywords:** China, mumps, transmissibility, age-specific dynamics, time-varying reproduction number, Jilin

## Abstract

**Objectives:**

Despite the adoption of a new childhood immunization program in China, the incidence of mumps remains high. This study aimed to describe the epidemiological characteristics of mumps in Jilin Province from 2005 to 2019 and to assess the transmissibility of mumps virus among the whole population and different subgroups by regions and age groups.

**Methods:**

The Non-age-specific and age-specific Susceptible–Exposed–Pre-symptomatic–Infectious–Asymptomatic–Recovered (SEPIAR) models were fitted to actual mumps incidence data. The time-varying reproduction number (*R*_*t*_) was used to evaluate and compare the transmissibility.

**Results:**

From 2005 to 2019, a total of 57,424 cases of mumps were reported in Jilin Province. The incidence of mumps was the highest in people aged 5 to 9 years (77.37 per 100,000). The two SEPIAR models fitted the reported data well (*P* < 0.01). The median transmissibility (*R*_*t*_) calculated by the two SEPIAR models were 1.096 (range: 1.911 × 10^−5^–2.192) and 1.074 (range: 0.033–2.114) respectively. The age-specific SEPIAR model was more representative of the actual epidemic of mumps in Jilin Province from 2005–2019.

**Conclusions:**

For mumps control, it is recommended that mumps-containing vaccines (MuCV) coverage be increased nationwide in the 5–9 years age group, either by a mumps vaccine alone or by a combination of vaccines such as measles-mumps-rubella (MMR) vaccine. The coverage of vaccines in Jilin Province should be continuously expanded to establish solid immunity in the population. China needs to redefine the optimal time interval for MuCV immunization.

## Introduction

With the introduction of two doses of measles, mumps, and rubella (MMR) vaccine (1) into national immunization program (NPI) in many countries, the incidence of mumps has declined dramatically (1). China is a country with a high number of reported mumps cases, and despite the provision of free routine single-dose MMR vaccination for children aged 18–24 months, since the implementation of the Expanded Programme on Immunization (EPI) in 2008, the incidence of mumps has not decreased significantly (2). In recent years, there have been an average of more than 250,000 cases of mumps infections in China each year, of which more than 90% are in children under 16 years of age, with children aged 5 and 9 years being at high risk for mumps (3–5). This situation is similar in other developed countries. For example, the high-risk population of the mumps outbreak in the United States between 2000 and 2015 was mainly concentrated in people aged 5–17 years (6). However, in many countries and regions that have been highly vaccinated, such as Portugal, Norway, Australia, etc. The number of cases has rebounded, mainly among adolescents and young adults, due to a combination of factors including waning vaccine immunity and opposition to vaccination (7–10).

Mumps virus (MuV) is the major cause of Mumps, but we have a limited understanding of the transmissibility of Mumps virus, which is responsible for the high incidence (140–726 per 100,000) (11, 12) for this vaccine-preventable disease. Many modeling studies have not only discussed and explained the relationship between mumps and meteorological factors, vaccination rates, etc. but have also predicted trends in mumps incidence in the coming years (13–16). These studies have led to a common view that the mumps epidemic remains serious and MMR vaccination coverage should be increased. A booster dose of MMR vaccine should be administered to high-risk groups such as students, and Anders Hviid et al. showed that the ideal schedule for mumps vaccination should be determined to optimize vaccine control and suggested that the optimal age for the second dose remains to be determined (17). Regarding to the current immunization strategy in China, many studies advocate adjusting the timing and doses of MMR vaccination to reduce the future incidence of mumps in China, but there is insufficient theoretical support (15, 18). The immunization schedules in China were adjusted after 2020 to one dose of MMR vaccine at 8 months of age and one dose at 18–24 months of age. However, there is evidence in the literature that MMR vaccine at shorter intervals may not reduce future trends in mumps incidence (10, 18). None of these studies explored the differences in the transmission of MuV across age groups, making it difficult to determine the optimal age for MuCV immunization due to the lack of a theoretical basis. The benefits of a new immunization policy for mumps are still unclear. Therefore, future rescheduling of MuCV in China may also be necessary. This study analyzed the daily incidence of mumps in Jilin Province from 2005–2019 and detailed the characteristics of epidemic changes between the whole population and different age groups during the 15-year period.

Two different Susceptible–Exposed–Pre-symptomatic–Infectious–Asymptomatic–Recovered (SEPIAR) models were created firstly to describe the epidemiology of mumps in the whole population of Jilin Province from 2005–2019 and to assess the differences of mumps transmissibility within and between different age groups to determine the optimal age for second or booster vaccination in Jilin Province, which can provide a reliable theoretical basis for the development of future MuCV immunization schedule intervals in China.

## Materials and methods

### Study design

In this study, a non-age-specific SEPIAR model and an age-specific SEPIAR model with seasonally adjusted parameters were developed using a mathematical epidemiological approach to characterize the transmissibility of mumps throughout the whole population and in different age groups.

### Data collection

We collected reported data of mumps in Jilin Province from 1 January 2005 to 31 December 2019 from the Chinese Information System for Disease Control and Prevention (CISDCP). Since 2004, as a Category C notifiable infectious disease, mumps should be reported through CISDCP within 24 h. The diagnosis of mumps is based on the mumps criteria established by the National Health and Well-ness Commission of the People's Republic of China. Data include information on gender, age, occupation, address, date of onset, and date of diagnosis. Population data were obtained from the Statistical Yearbook.

## Model construction

### Non-age-specific SEPIAR model

**Figure 1:**
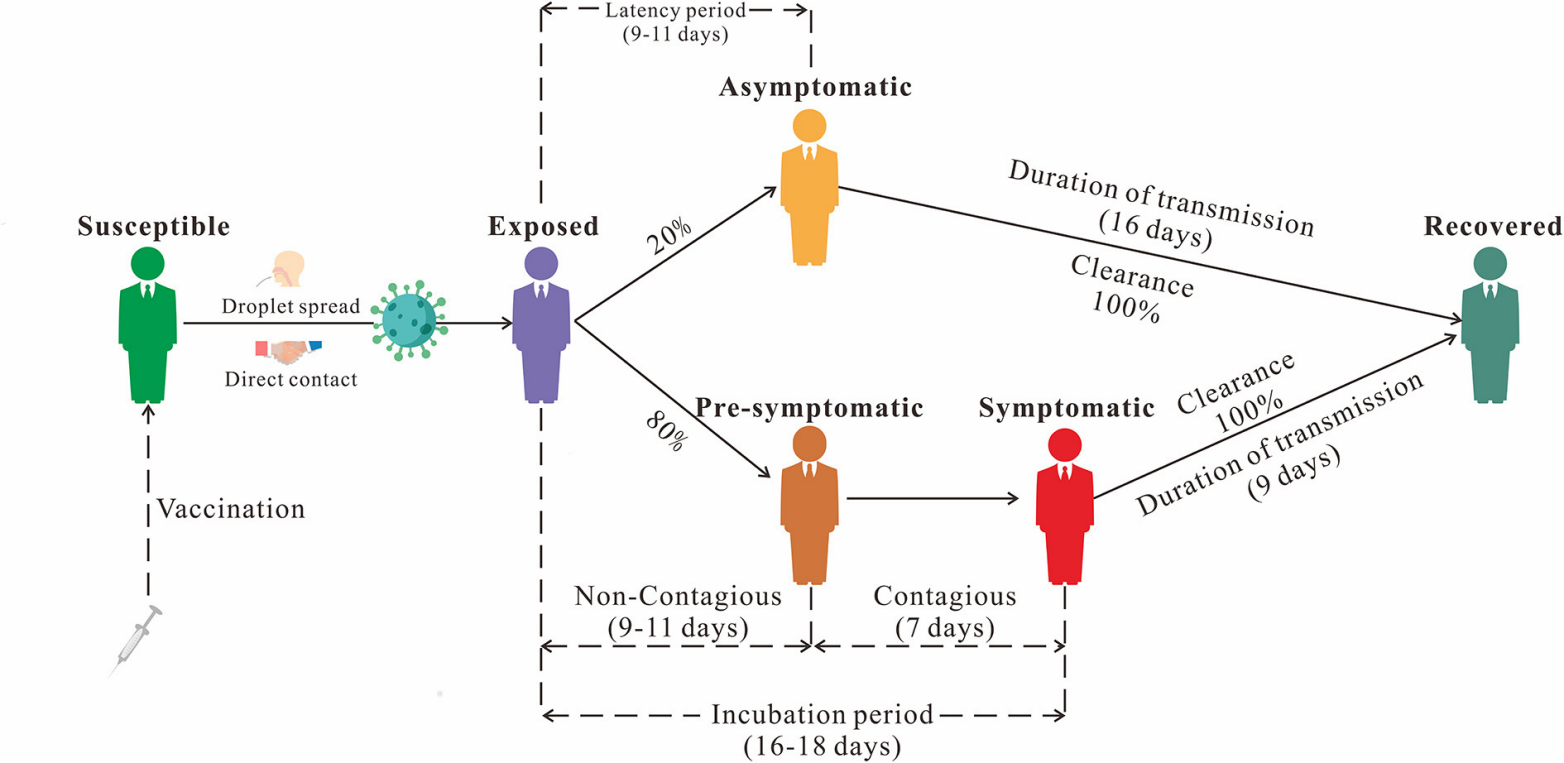
The natural history framework of mumps.

We established the non-age-specific SEPIAR model with the addition of compartment *P* (Pre-symptomatic) based on our previous SEIAR model (19). In this model, the population is divided into six categories based on the natural history of mumps: susceptible (*S*), exposed (*E*), pre-symptomatic (*P*), infectious (*I*), asymptomatic (*A*), and recovered/removed (*R*) (Figure 1). This model was based on the following assumptions:

(1) Set *n* as the number of population, *b*_*r*_ as the birth rate, and *d*_*r*_ as the natural death rate.
(2) Suppose that the infection rate coefficient after effective contact between *S* and *I* is β, and that asymptomatic *A* and pre-symptomatic *P* are infectious and their transmissibility *k*_1_ (0 ≤ *k*_1_ ≤ 1) and *k*_2_ (0 ≤ *k*_2_ ≤ 1) times that of Infectious *I*, then at *t* moment, the number of new infections is β*S* (*I* + *k*_1_*A*+ *k*_2_*P*).
(3) The proportion of asymptomatically infected was defined as ρ, the exposed individuals become asymptomatic and pre-symptomatic cases after an incubation period (1/ω_1_) and a latent period (1/ω_2_). the numbers of people who change from *E* to *A* and *P* at time *t* are ρω_1_*E* and (1-ρ) ω_2_*E*, respectively. the number of people who change from *P* to *I* at *t* time is ω_3_*P*.
(4) The asymptomatic and symptomatic cases are transferred into removed persons after an infectious period of 1/γ and 1/γ_1_, respectively.

This model equation is a special case of the following age-specific SEPIAR model equation, so it will not be repeated here.

### Age-specific SEPIAR model

Considering the interaction between different age groups, we create an age-specific SEPIAR model. We divide the total population into five age groups and use the subscripts *i* and *j* to denote age groups 1 to 5. (*i* ≠ *j*; 1: 0–4 years old; 2: 5–9 years old; 3: 10–14 years old; 4: 15–19 years old and 5: ≥ 20 years old). Mumps can be transmitted within each age group, so the transmission rate within a given age group *i* is denoted as β_*ii*_. Mumps can be transmitted between different age groups, so the transmission rate from age group *i* to *j* is β_*ij*_ and that from *j* to *i* is β_*ji*_.

**Figure 2:**
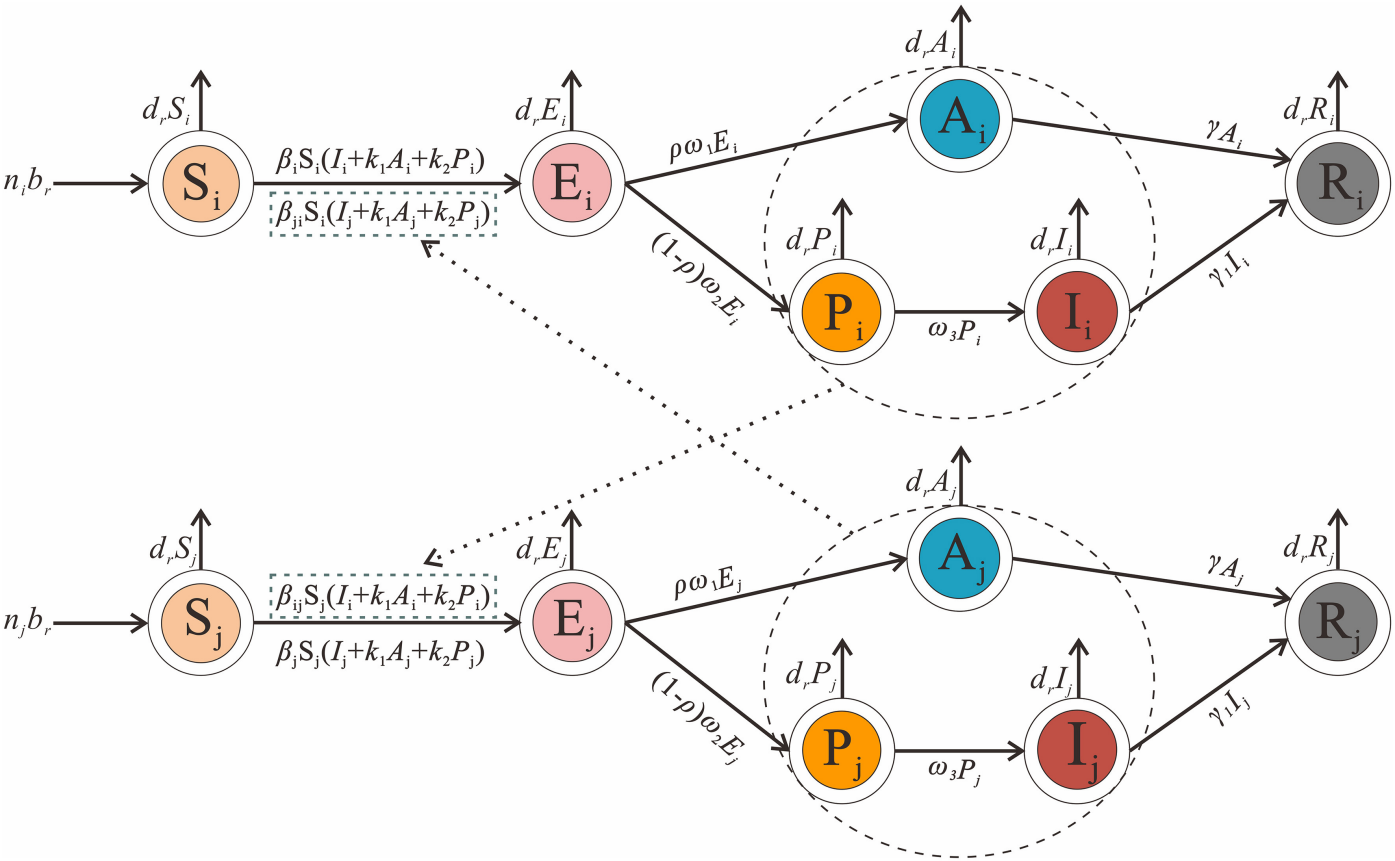
Age-specific SEPIAR model framework of mumps. [*i* and *j* represent age 0–4, 5–9, 10–14, 15–19 and ≥20, and respectively (*i* ≠ *j*)].

The model framework is shown in Figure 2. The equations of the model are shown as follows:


$$\begin{array}{l} dS_{i}/dt\;=\;Nb_{r}-S_{i}\overset{n}{\underset{j=1}{\sum^{\text{​}}}}\beta_{ji}(I_{j}+k_{1}A_{j}+k_{2}P_{j})-d_{r}S_{i}\\ dE_{i}/dt\;=\;S_{i}\overset{n}{\underset{j=1}{\sum^{\text{​}}}}\beta_{ji}(I_{j}+k_{1}A_{j}+k_{2}P_{j})-\rho \omega_{1}E_{i}\\ \;-\;(1-\rho )\omega_{2}E_{i}-d_{r}E_{i}\\ dP_{i}/dt\;=\;(1-\rho )\omega_{2}E_{i}{}_{-}\omega_{3}P_{i}-d_{r}P_{i}\\ dI_{i}/dt\;=\;\omega_{3}P_{i}-\gamma_{1}I_{i}-d_{r}I_{i}\\ dA_{i}/dt\;=\;\rho \omega_{1}E_{i}-\gamma A_{i}-d_{r}A_{i}\\ dR_{i}/dt\;=\;\gamma_{1}I_{i}+\gamma A_{i}-d_{r}R_{i}\\ \;i\;=\;1,2,\ldots ,n \end{array}$$

### Seasonality of transmission

In this study, we considered the seasonality of transmission. The Seasonality should be dynamic, with a focus on β. Therefore, the following trigonometric functions were applied as follows:


$$\begin{array}{l} \beta =\beta_{0}\left[1+sin\left(\frac{2\pi \left(t+\alpha \right)}{T}\right)\right] \end{array}$$

In this equation, β_0_, *t*, α and *T* refer to the baseline of the relative rate of transmission, the time, the constant to adjust the time position and the time span of the seasonal cycle.

### Parameter estimation

According to the two model and the natural history framework of mumps, there are eight parameters involved: birth rate *b*_*r*_, natural mortality rate *d*_*r*_, infection coefficient without age groups β, infection coefficient by age groups β_*ij*_ (the subscripts *i* and *j* (*i* ≠ *j*) represent age groups 1 to 5), proportion of the asymptomatic ρ, latent relative rate ω_1_, non-contagious relative rate ω_2_, contagious relative rate ω_3_, and the recovery removal rate coefficient γ and γ_1_. A systematic review has further confirmed that the proportion of asymptomatic mumps is between 15 and 27%, and it is suggested that the ρ should be set at 0.2 (20, 21). The incubation period of mumps is 16–18 days (22). As mumps patients can secrete MuV from saliva approximately 1 week before the onset of salivary gland enlargement to 9 days after the onset, the contagious and recovery periods of mumps patients are 7 and 9 days respectively, so ω_3_ = 1/7 and γ_1_ = 1/9. The value were 0.14286 and 0.11111, respectively (17, 23–25). The latent period and non-contagious of mumps were the difference between the incubation period and the contagious period, which were 9–11 days. This value was 10 days, so ω_1_ = ω_2_ =1/10. The value was 0.1. Combining the actual epidemic definition of mumps in Jilin Province with a previous study (19), the disease course range was set to 5–25 days, with a value of 16 days and γ = 1/16. The value was 0.06250. In addition, the value of the parameter *k*_1_ and *k*_2_ are set to 0.3 (19). The significance and estimation of each parameter and the initial value settings of the variables are shown in Table 1.

**Table 1:** Descriptions and values of parameters in the two models of mumps.

**Parameter**	**Descriptions**	**Method**	**Unit**	**Range**	**Value**
*b_*r*_*	Birth rate	Analysis on the reported data	years^−1^	0–1	0.00662
*d_*r*_*	Mortality rate	Analysis on the reported data	years^−1^	0–1	0.00551
β	Transmission relative rate	Curve fitting	days^−1^	0–1	–
*β_*ii*_*	Transmission relative rate within age group *i*	Curve fitting	days^−1^	0–1	–
*β_*ij*_*	Transmission relative rate from age group *i* to *j*	Curve fitting	days^−1^	0–1	–
*β_*ji*_*	Transmission relative rate from age group *j* to *i*	Curve fitting	days^−1^	0–1	–
*β_*jj*_*	Transmission relative rate within age group *j*	Curve fitting	days^−1^	0–1	–
*k*_1_	Relative transmissibility rate of asymptomatic to symptomatic individuals	Reference	1	0–1	0.3
*k*_2_	Relative transmissibility rate of pre–asymptomatic to symptomatic individuals	Reference	1	0–1	0.3
ρ	Proportion of the asymptomatic	Reference	1	0.15–0.27	0.2
ω_1_	Latent relative rate	Reference	days^−1^	0.09–0.11	0.1
ω_2_	Non–contagious relative rate	Reference	days^−1^	0.09–0.11	0.1
ω_3_	Contagious relative rate	Reference	days^−1^	–	0.14286
γ	Removed rate of the asymptomatic	Reference	days^−1^	0.04–0.2	0.06250
γ_1_	Removed rate of the infections	Reference	days^−1^	0.04–0.2	0.11111

### Quantitative evaluation of transmissibility

According to previous studies (19, 26, 27), the transmissibility of mumps virus is quantified by the basic reproduction number (*R*_0_), *R*_0_ has a threshold characteristic that if *R*_0_ < 1, each infected individual produces on average < 1 secondary case during their lifetime and therefore the epidemic fades away, whereas if *R*_0_ > 1, the disease may result in a large number of new infections. Estimating the correct *R*_0_ is crucial in an epidemic emergency response, as it provides direction for subsequent control measures. When the intervention is applied, *R*_0_ is expressed as the effective reproduction number (*R*_*eff*_) which reflects the real transmissibility of infectious diseases (26, 27). The effectiveness of prevention and control measures can be evaluated by calculating the *R*_*eff*_ value for different time periods and comparing this value with the change in the threshold value for different time periods. In this study, we first calculate *R*_*eff*_ and then obtain the time-varying reproduction number (*R*_*t*_) based on the time-varying (*S*_(t)_, β_(t)_, …) in the *R*_*eff*_ expression. Therefore, we used *R*_*t*_ to assess the transmissibility of mumps. Due to the optimization of the compartments and parameters involved in this research model compared to the previous model, the next-generation matrix method (NGM) was used in this study to calculate *R*_*t*_ (28). The calculation procedure is detailed in Additional file 1.

### Simulation method and statistical analyses

We used the MATLAB (R2021a) and Berkeley Madonna 8.3.18 software (developed by Robert Macey and George Oster of the University of California at Berkeley. Copyright1993–2001 Robert I. Macey & George F. Oster) to model the actual situation of mumps in Jilin province. The simulation methods (Runge–Kutta method of order four with tolerance set to 0.001) were the same as those used in previously published studies (29–33). The models were used to fit the data to calculate the parameters. The transmissibility was calculated according to the parameters. The goodness of fit of mumps in Jilin province was calculated using the coefficient of determination (*R*^2^) values and *P*-values in SPSS 21.0 (IBM Corp, Armonk, USA). If *R*^2^ ≥ 0.5 and *P* < 0.05, the models fitted well and the results were statistically significant, if *R*^2^ < 0.5 or *P* > 0.05, the models did not fit well or the results were not statistically significant.

## Results

### Epidemiological description of mumps in Jilin Province

**Figure 3:**
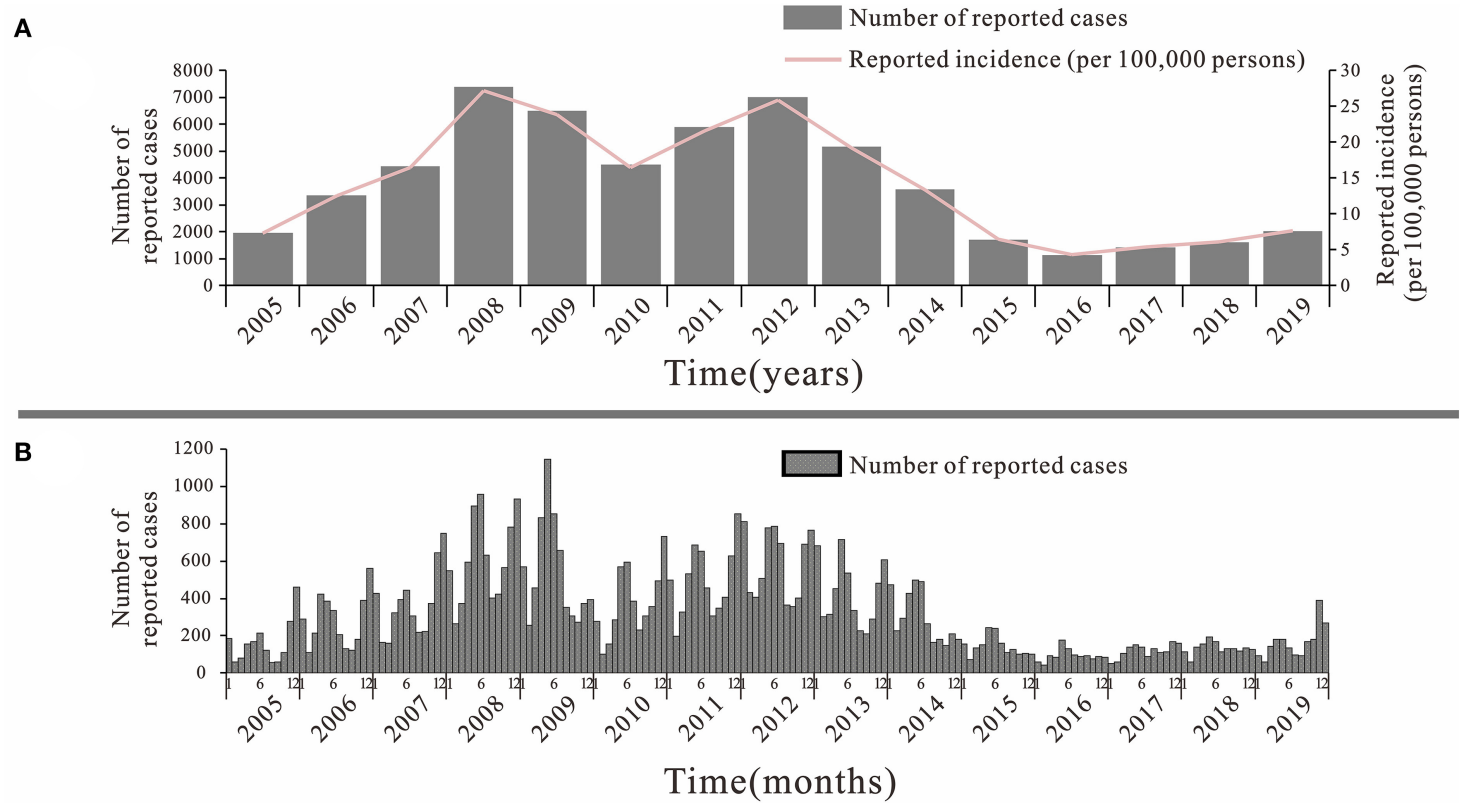
Mumps cases and incidence rate in Jilin Province, 2005–2019. **(A)** By years. **(B)** By months.

From 2005 to 2019, a total of 57,424 cases of mumps were reported in Jilin Province, with an overall decreasing trend (Figure 3A). The lowest incidence rate was 4.26 per 100,000 in 2016 and the highest was 27.18 per 100,000 in 2008. There were two large increases during this 15-year period, the first from 2005 to 2008 and the second from 2010 to 2012, with the first peak being higher than the second. The incidence of mumps showed a decreasing trend from 2012 to 2016, followed by a slow increase until 2019. The trend in mumps incidence by month from 2005–2019 shows that there are two peaks intervals of mumps incidence in each year, namely May-July and around November and January of the following year, with a clear seasonal trend, but this trend is not obvious after 2015 (Figure 3B).

**Figure 4:**
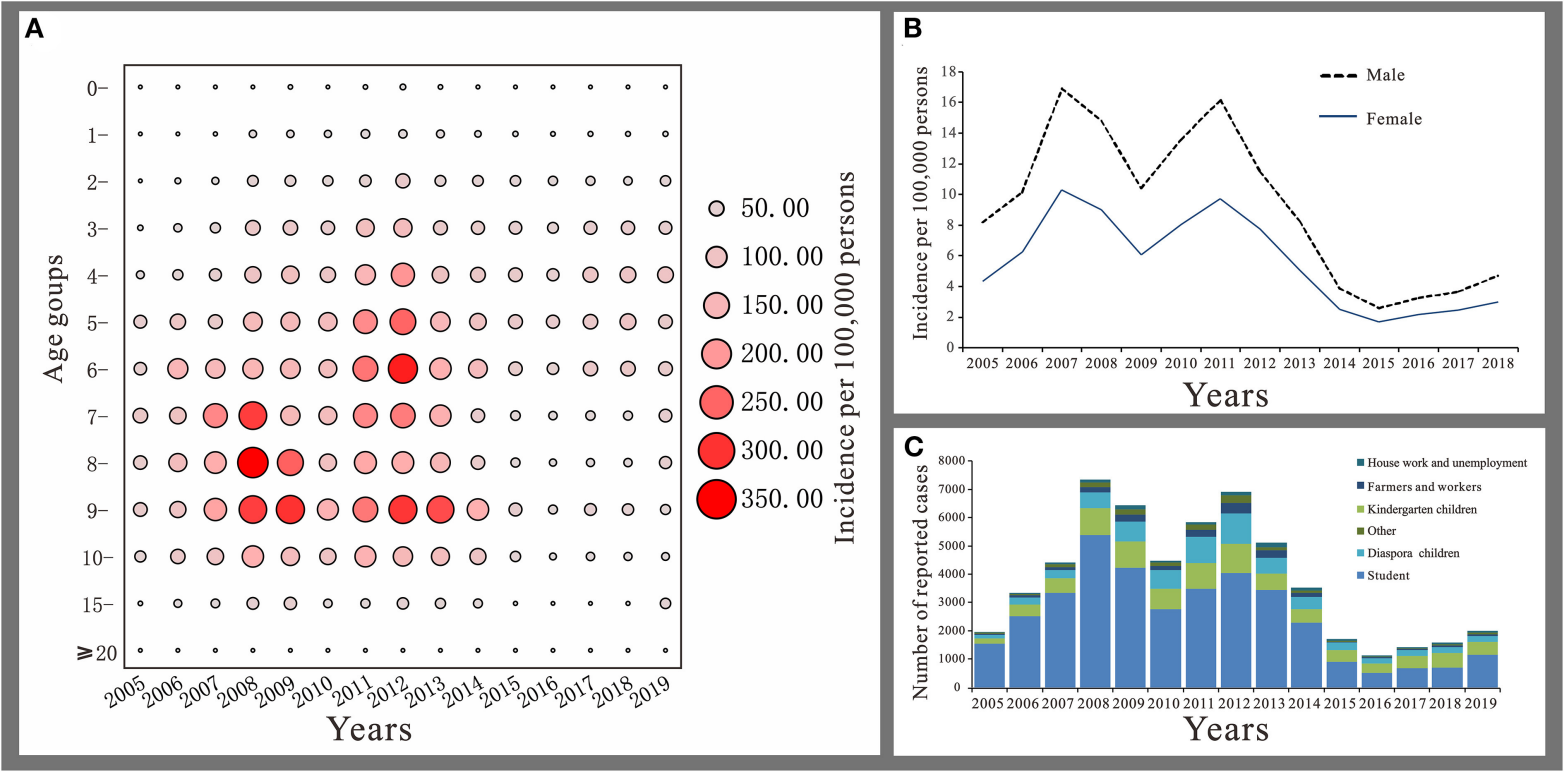
Distribution of mumps in Jilin Province. **(A)** Age distribution. **(B)** Gender distribution. **(C)** Occupation distribution.

The reported cases of mumps mainly involved patients aged 3 to 14 years, accounting for 75.44% of the total number of cases. Patients aged 5 to 9 years had the highest average annual incidence rate of 77.37 per 100,000 and patients <1 year had the lowest number of cases accounting for only 0.28% of the total number of cases, with an average annual reported incidence rate of 0.53/100,000 (Figure 4A). Among all cases, there were 35,746 males with an average annual reported incidence rate of 8.85 per 100,000. 21,726 females with an average annual reported incidence rate of 5.38/100,000. The ratio of males to females incidence rate was 1.65:1 (Figure 4B). Students accounted for the highest proportion of total cases (64.27%). Except from 2016 to 2018, students accounted for more than 50% of the total cases, followed by kindergarten children and diaspora children, with a cumulative number of cases of 8,831 and 6,647 cases, accounting for 15.37 and 11.57% of the total cases, respectively (Figure 4C).

**Figure 5:**
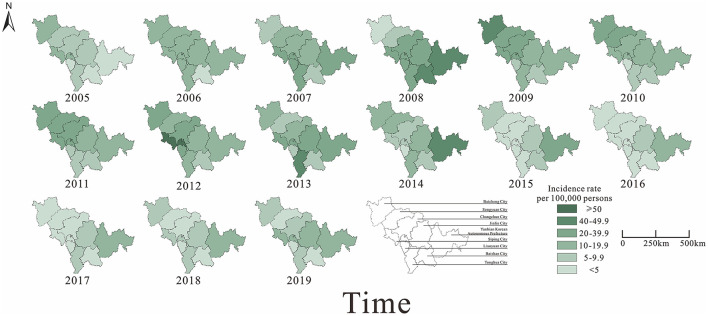
Map of the incidence rate of mumps in Jilin Province, 2005–2019.

From 2005 to 2019, a total of 57,375 cases of mumps were reported in nine cities in Jilin Province. Of these, Changchun City (15,279 cases) had the highest number of cases compared to other cities, while Baishan City (1,535 cases) and Liaoyuan City (1,508 cases) had the lowest number of cases. The trends of the epidemic shifted from intermediate urban areas to neighboring urban areas from 2005 to 2008. The incidence of mumps was at a high level in all cities of Jilin province after 2008, and decreased gradually after 2014. Incidence rates in neighboring urban areas started to decrease later than in intermediate urban areas and started to decrease significantly from 2015. Since 2016, incidence rates have been increasing in all districts, but not to a significant extent. Overall, the average annual incidence rates were relatively high in Siping City and Yanbian Korean Autonomous Prefecture, while lower in Baishan City and Liaoyuan City (Figure 5).

### Models fitting results and evaluation of effectiveness

**Figure 6:**
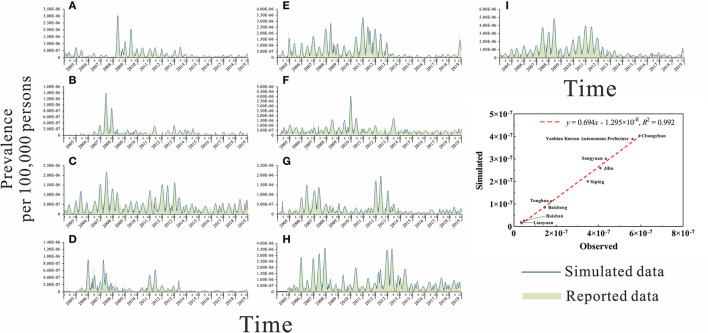
Fitting results of mumps cases in 9 cities of Jilin Province using the non-age-specific SEPIAR model. **(A)** Baicheng City. **(B)** Baishan City. **(C)** Jilin City. **(D)** Liaoyuan City. **(E)** Siping City. **(F)** Songyuan City. **(G)** Tonghua City. **(H)** Yanbian Korean Autonomous Prefecture. **(I)** Changchun City.

Firstly, the actual prevalence of mumps in nine cities in Jilin Province from 2005–2019 was fitted using the non-age-specific SEPAIR model (Figure 6). The results were good and were statistically significant in all cities (*R*^2^ > 0.6, *P* < 0.01), with the best fit effect in Baicheng City (*R*^2^ = 0.87, *P* < 0.01) (Table 2).

**Table 2:** Goodness–of–fit test of nine cities in Jilin Province.

**City**	***R*** **^2^**	***P***
Baicheng City	0.87	< 0.01
Baishan City	0.76	< 0.01
Jilin City	0.69	< 0.01
Liaoyuan City	0.77	< 0.01
Siping City	0.86	< 0.01
Songyuan City	0.78	< 0.01
Tonghua City	0.83	< 0.01
Yanbian Korean Autonomous Prefecture	0.86	< 0.01
Changchun City	0.85	< 0.01

**Figure 7:**
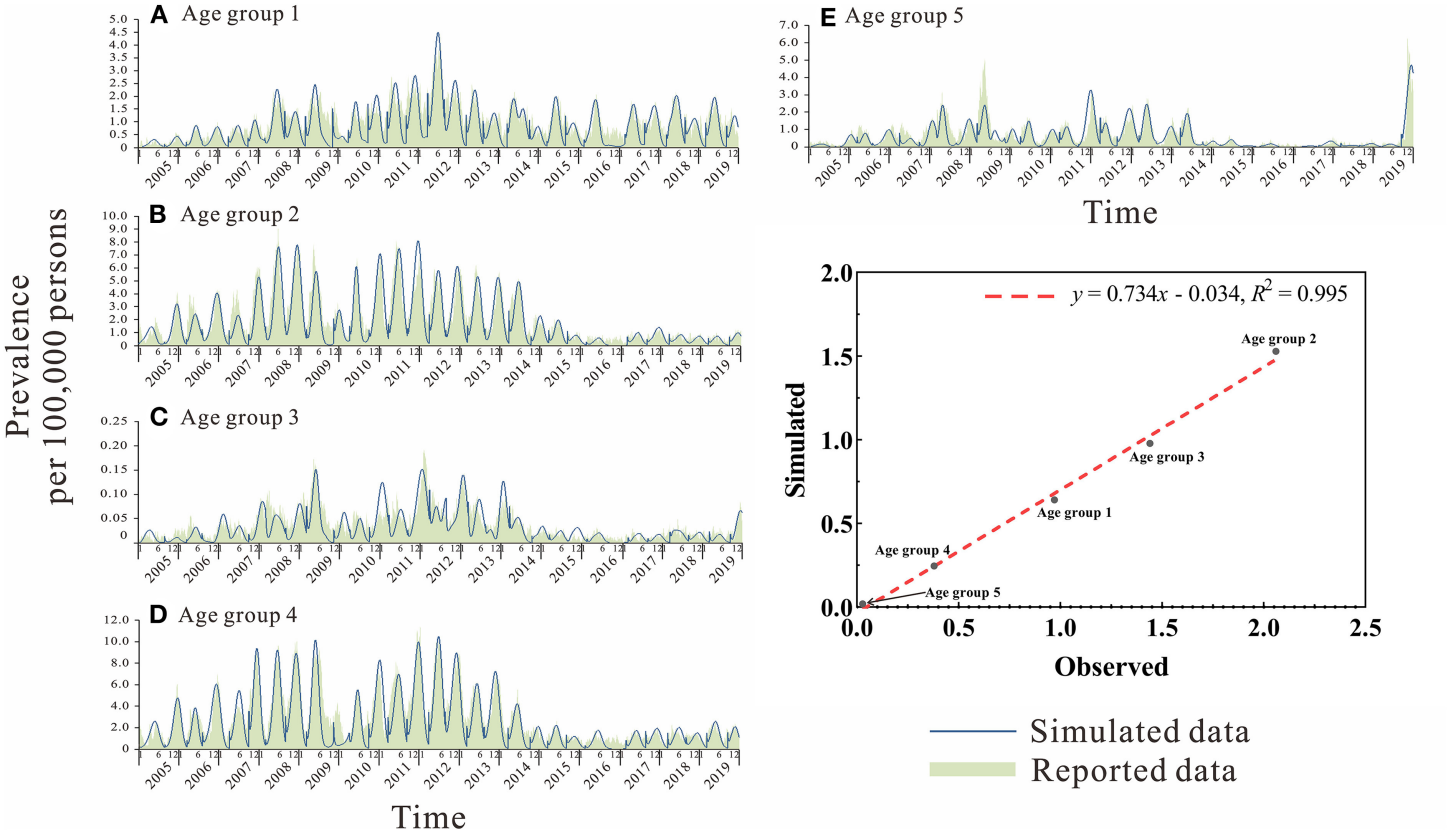
Curve fitting of the age-specific SEPIAR model to the reported data of different age groups in Jilin Province. **(A)** Age group 1: 0–4 years. **(B)** Age group 2: 5–9 years. **(C)** Age group 3: 10–14 years. **(D)** Age group 4: 15–19 years. **(E)** Age group 5: ≥ 20 years.

Secondly, the age-specific SEPAIR model was used to fit the actual prevalence of mumps in different age groups (Figure 7). The best-fit were obtained for age group 2 (5–9 years old) and age group 3 (10–14 years old) (*R*^2^ > 0.8, *P* < 0.01) (Table 3).

**Table 3:** Goodness–of–fit test of each age group.

**Age groups**	***R*** **^2^**	***P***
0–4 years old	0.62	< 0.01
5–9 years old	0.81	< 0.01
10–14 years old	0.8	< 0.01
14–19 years old	0.71	< 0.01
≥ 20 years old	0.63	< 0.01

### Assessment of transmissibility

**Figure 8:**
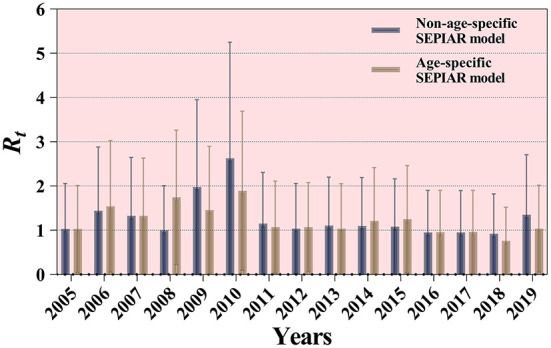
Results of two SEPIAR models for the calculation of the transmissibility of mumps in Jilin Province, 2005–2019.

The non-age-specific SEPAIR model and the age-specific SEPAIR model were used to calculate the transmissibility of mumps in Jilin Province from 2005 to 2019, respectively. The results showed that the overall median *R*_*t*_ was 1.096 (range: 1.911 × 10^−5^–2.192) and 1.074 (range: 0.033–2.114), respectively, during these 15 years. The median *R*_*t*_ for mumps calculated by both models was > 1 except in 2006 and 2018, with the highest in 2010 at 2.625 (range: 2.425 × 10^−6^–5.251) and 1.894 (range: 0.097–3.691), respectively (Figure 8).

**Figure 9:**
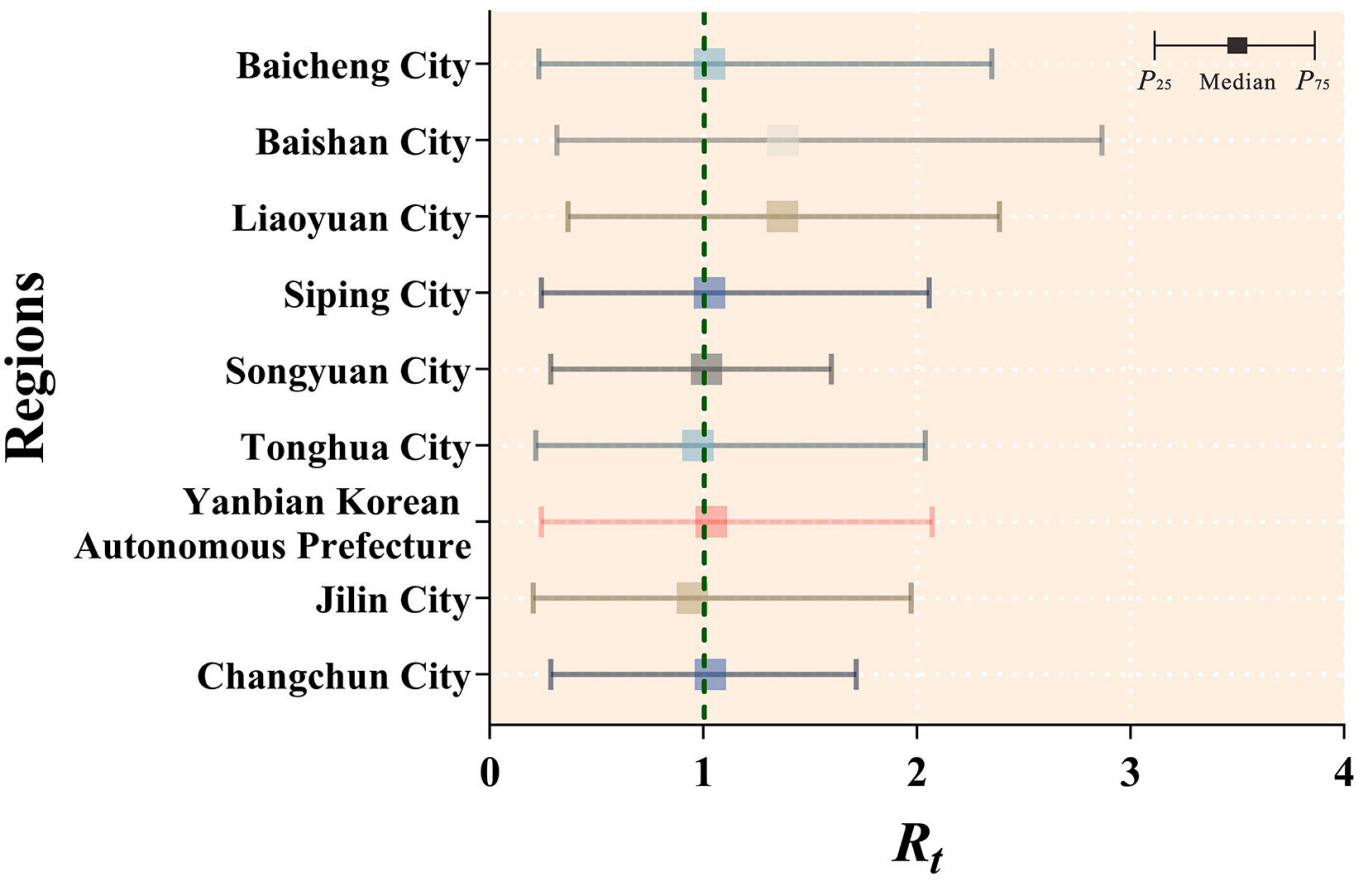
Calculation results of transmissibility of mumps in 9 cities in Jilin Province.

Based on a non-age-specific SEPAIR model, the transmissibility of mumps was calculated for nine cities in Jilin Province from 2005 to 2019, where the median *R*_*t*_ larger than 1 was Changchun City (median = 1.033, IQR: 0.286–1.716); Yanbian Korean Autonomous Prefecture (median = 1.037, IQR: 0.241–2.072); Songyuan City (median = 1.015, IQR: 0.287–1.600); Siping City (median = 1.030, IQR: 0.243–2.058); Liaoyuan City (median = 1.370, IQR: 0.367–2.386); Baishan City (median = 1.373, IQR: 0.313–2.866); Baicheng City (median = 1.029, IQR: 0.230–2.350). Only two cities had median *R*_*t*_ values < 1, namely Jilin City (median = 0.950, IQR: 0.204–1.972) and Tonghua City (median = 0.976, IQR: 0.216–2.039) (Figure 9).

**Figure 10:**
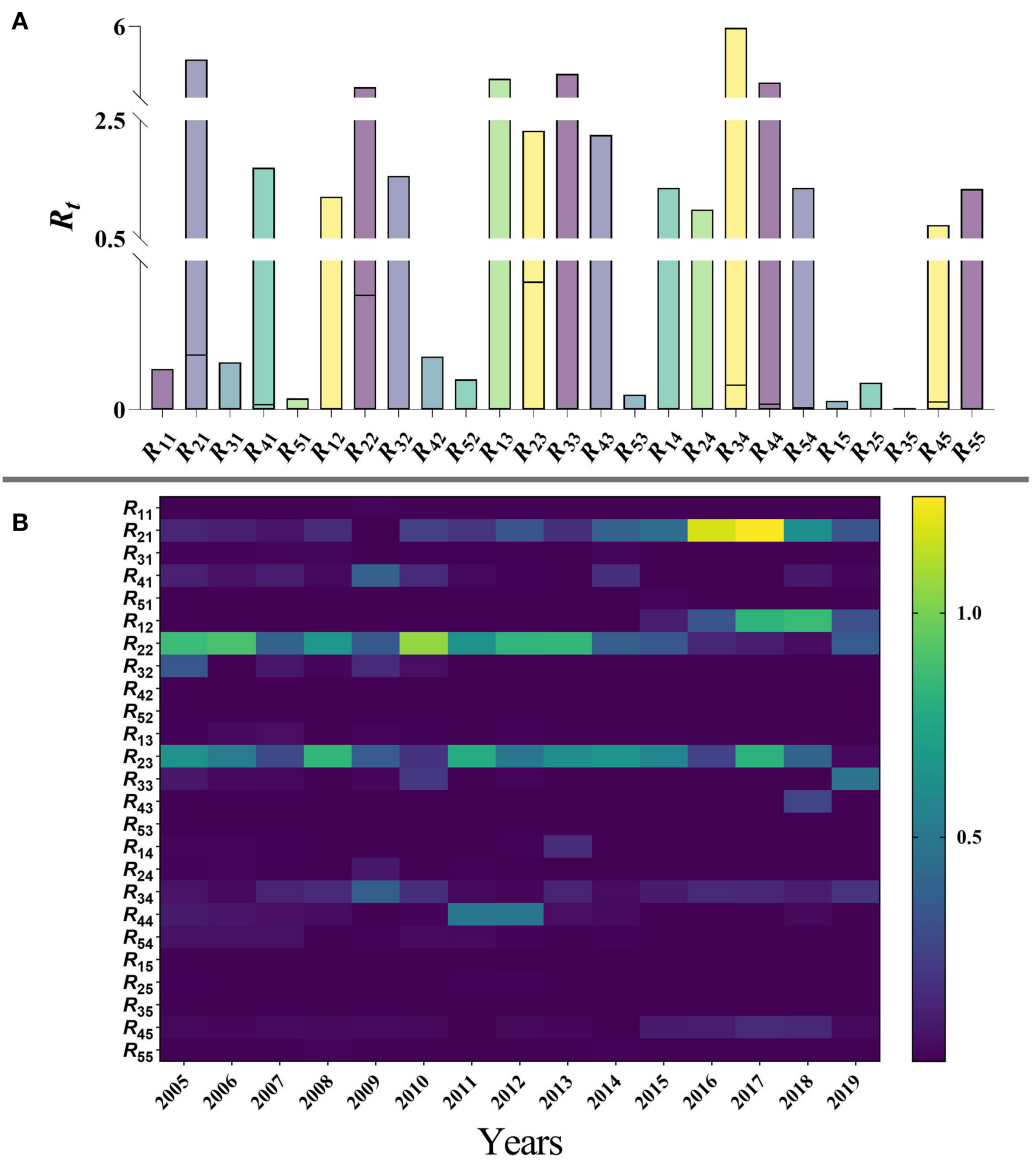
**(A)** Range of relative transmissibility by five age groups of Jilin Province. **(B)** Calculation results of relative transmissibility of five age groups of mumps in Jilin Province, 2005–2019. (1: 0–4 years; 2: 5–9 years; 3: 10–14 years; 4: 15–19 years; 5: ≥ 20 years).

Based on the age-specific SEPAIR model, the results of the *R*_*ij*_ values of mumps within and between each age group (with *i* and *j* used to represent age groups 1 to 5) in Jilin Province during 2005–2019 are as follows: the highest transmission risk occurred from patients in age group 2 to patients in age group 3 (median = 0.428, range: 1.519 × 10^−7^–2.321), namely age group 2 to 3, followed by age group 2 to 2 (median = 0.384, range: 8.310 × 10^−8^–3.029) and age group 2 to 1 (median = 0.184, range: 2.770 × 10^−9^–4.384); the lowest transmissibility occurred from age group 3 to 5 (median = 6.166 × 10^−9^, range: 5.739 × 10^−21^–3.314 × 10^−3^), followed by age group 3 to 2 (median = 4.854 × 10^−8^, range: 2.080 × 10^−18^-1.562) and age group 5 to 1 (median = 5.130 × 10^−8^, range: 2.744 × 10^−19^–3.762 × 10^−2^). The median relative transmissibility showed that the highest transmission was from patients in group 2 to patients in group 3, followed by age group 2 to 2 and age group 2 to 1. The maximum value of the relative transmissibility showed that the relative transmission within other age groups is > 1 except within age group 1 (Figure 10A). Over the 15-year period, the transmissibility of age Group 2 to 2 varied significantly, with fluctuating trends before 2010, then reached a peak in 2010 (the median of *R*_*t*_ = 1.052, range: 1.82910^−5^–3.020) and then decreased each year. Secondly, the transmissibility of age groups 2 to 3 peaked every 4 years before 2011, declines to zero after 2011, but reached a peak in 2017 (median = 0.806, range: 5.03110^−6^–1.079). It is noteworthy that age group 2 to 1 had a high transmissibility in both 2016 (median = 1.182, range: 6.532 × 10^−6^–4.142) and 2017 (median = 1.260, range: 2.304 × 10^−5^–4.384) respectively (Figure 10B).

## Discussion

In this study, an age-specific SPEIAR model was used to assess the transmissibility of mumps, considering the population heterogeneity in the transmission. This model takes into account differences in the transmission of MuV between and within age groups, more accurately assessed the transmissibility of mumps in different age groups, and better identified the age of high mumps incidence. The results of the goodness-of-fit test imply that the validity of the models is high and that the two models accurately reflect the real situation of mumps in Jilin Province from 2005-2019.

During 2005–2019, the overall median transmissibility of mumps was > 1 in all cities of Jilin Province, indicating a natural epidemic trend of mumps throughout Jilin province during this 15 years period, which is consistent with the situation in China (4). The incidence of mumps in Jilin Province has a cyclical trend with outbreaks every 4–5 years and two peak months per year (June and December), which is consistent with previous results (34, 35). In China, MuCV was not included in non-pharmaceutical interventions until 2008 because the voluntary, self-funded vaccination requirement resulted in very low national coverage. Since 2008, routine single-dose MMR vaccination has been offered to children aged 18–24 months (14). However, the incidence of mumps in Jilin Province did not decrease significantly in 2008–2010 and was slightly higher than that in 2005–2007. This observation may be attributed to the persistently low vaccination rate. However, despite China's push for annual MMR vaccine coverage of more than 95 % since 2009 (2), mumps in Jilin Province was not effectively controlled and peaked again in 2012. This suggests that a single dose of MMR vaccine has limited efficacy in containing the spread of mumps (36). Even children who receive a single dose of MMR vaccine have declining levels of mumps antibodies as they grow older and become susceptible to mumps again (37).

Mumps outbreaks often occur in densely populated areas such as kindergartens, schools, military camps, communities, etc (35). The literature on epidemiology has found a significantly higher incidence in males than in females, which is consistent with our study and may be due to the behavioral patterns of males and females, with boys being more active and more likely to have increased exposure to MuV than girls (2, 38–40). In this study, the highest incidence rates of mumps were found in the 5–9 years age group. This may be a direct consequence of susceptibility to mumps in children aged 5–9 years who were not vaccinated with MuCV. Children who were vaccinated at 18–24 months of age may be more susceptible to mumps virus infection during primary school (41, 42). The peak months of mumps outbreaks in Jilin Province coincide with school semesters, and the closed environment and management of schools facilitate the spread of mumps, which is consistent with our findings that students are a high-risk occupation for mumps (43). Therefore, students are a priority population for prevention, and it is recommended to check vaccination registrations before school entry, provide booster vaccinations to unvaccinated students, and strengthen school health education.

There are a number of studies that have used *R*_0_ to estimate the transmissibility of mumps. The *R*_0_ was 3.6–4.5 in many European countries from 1970 to1990 (44). Kanaan et al. estimated the *R*_0_ for mumps was 19.3 using a matrix model with individual heterogeneity in the UK in 1986 (45). the *R*_0_ of mumps was about 4.3 in China from 2009 to 2015 (14). In 2012 and 2014 years, the *R*_0_ for children in schools was 4.49 and 2.50 in Shandong, China, respectively (46). In contrast, the median value of *R*_0_ for mumps estimated by our model was slightly > 1, which was much lower than the results of the above studies. We think this is mainly related to the continued EPI for children in Jilin Province after 2008, which has increased the MMR vaccination rate. This has resulted in a decrease in the susceptible population year by year. Those who have been previously vaccinated are partially immune, so MuV transmission is less likely to occur in the population and subsequent infection rates are low. The remaining reason is that, based on the description of high-risk occupations for mumps in Jilin Province, it cannot be ruled out that mumps outbreaks are highly likely to occur in schools even with MMR vaccination. Therefore, we should be more concerned about the transmission characteristics of mumps in students.

The inconsistency in MMR vaccination rates is due to differences in socioeconomic status and health care conditions across cities. The incidence of mumps is lower in relatively developed areas, such as Jilin and Changchun, where better health care is available. In some areas with low socio-economic status and inadequate health services, such as Baishan City and Liaoyuan City, the transmissibility of MuV is high, which may be due to untimely mumps vaccination and low coverage. The proximity of different provinces and countries in Yanbian Korean Autonomous Prefecture, Baicheng City and Siping City and the large movement of people who have not acquired basic immunity against mumps, had led to large differences in the incidence of mumps in these three cities, suggesting that large movement of people across regions may also be a factor in the spread of mumps (7, 47). Therefore, it is recommended that MuCV coverage be continuously expanded nationwide to establish solid immunity among the entire population in order to eliminate mumps outbreaks in different regions.

We found that the transmissibility of mumps in Jilin Province showed a rapid increase until 2010 but a rapid decline in 2011, followed by low levels of stability with small fluctuations in the following years. The reason for this analysis was the use of MuCV as a class II vaccine before 2008, resulting in a significant under-coverage of MuCV. Even though China introduced a routine MMR vaccination program in 2008, the short duration of the introduction of the MMR vaccine did not recommend solid immunity in the population, resulting in a high level of transmissibility of mumps in Jilin Province until 2010. However, as MMR vaccine coverage increased year by year, the level of mumps antibodies in the population also increased, leading to a rapid decrease in the transmissibility of mumps in Jilin Province in 2011. Combined with the timing of peak mumps incidence and population distribution described previously, it is suggested that most mumps outbreaks in Jilin Province occurred in schools, which is the main reason for the stable trend and low fluctuation of mumps transmissibility after 2011. Therefore, we recommend that primary school students who have not received a booster dose of MMR be screened for missed vaccines.

Analysis of the relative transmissibility within and between different age groups showed high transmissibility in all age groups except 0–4 years. This suggests that frequent contact within different age groups could also cause outbreaks (48). In 2008, Remarkably, the study also found a high relative transmission contribution of the 5–9 years age group to the other age groups (except 14–19 years, ≥20 years) and within them. This suggests that the 5–9 years susceptible group was the main cause of the mumps epidemic in Jilin province during the 15 years period. In other Chinese provinces and cities, such as Beijing, Tianjin and Shanghai, the incidence of mumps was significantly lower after the second dose of the MMR vaccine was administered to children aged 4, 5 and 6 years, suggesting the effect of immune failure in the 5–9 years age group (40, 49). Due to the unbalanced development of economic and medical resources in China, the coverage of 1 dose of MMR vaccine is low in many places, not to mention the second dose of MMR vaccine, let alone a second dose of MMR vaccine, is low in many places; therefore, timely administration of a second dose or booster dose of MuCV after a single dose of vaccine is essential, depending on the decline of antibodies and the prevalence of the different age groups. Although the immunization schedule for MuCV in China has been adjusted in recent years to provide one dose of MMR vaccine for children 8 months of age and one dose for children 18 months of age (50), the previous study suggests that completing two doses of MMR vaccine in a shorter interval may lead to an increased risk of mumps infection in adolescents due to weakened immunity, and vaccination of preschool children seems more feasible (7).

Therefore, it is recommended that the MMR immunization strategy in Jilin Province be adjusted to set the optimal age for the second dose of MMR vaccine at 5–9 years of age and that it be expanded nationwide. It is of concern that if a new MMR vaccination schedule is developed as we envision, it is unknown whether the future epidemiological trend in China will result in mumps outbreaks in adolescents and young adults as in the United States and France (35). Therefore, as an alternative, in addition to the routine 2-dose of MMR vaccination in Jilin province according to the NPI, a booster vaccination with MuCV vaccine could be administered only to special groups of students before different school entry stages (elementary, middle, high school and university) to reduce the incidence of future mumps. As China updates its childhood NPI in 2021, the benefits of the new mumps immunization strategy are unclear at this stage, so we still need to observe and study future mumps surveillance data and seroepidemiology to provide a solid theoretical basis for developing an optimal interval for future MuCV immunization program in China.

There are several limitations that may have influenced the results obtained. To address the transmission characteristics of mumps in Jilin Province, we proposed the optimal age for implementing the second dose of MMR intervention, but did not specifically assess whether implementing this vaccine intervention at the optimal age could affect the epidemiological trend of mumps in Jilin Province at this stage. Therefore, we will develop age-specific SVEPIAR models under additional MuCV interventions to determine changes in mumps transmissibility and future epidemiological trends under different age-specific vaccine interventions in a comparative study to further provide stronger evidence for future MuCV immunization schedules in China.

## Conclusions

The epidemic pattern of mumps in Jilin Province from 2005–2019 was characterized by cyclical and seasonal epidemics, with peaks every 4 years and the highest incidence periods in May-July and November-January. The highest incidence was among children aged 5–9 years. The age-specific SEPIAR model more accurately reflects the true picture of mumps epidemic trends in Jilin Province from 2005–2019. The transmission risks were high from the 5–9 years age group to the 10–14 years age group and between the 5–9 years age group. The priority population for prevention is students by checking the vaccination registration before the start of school for timely replenishment. The coverage of vaccines in Jilin Province should be continuously expanded to establish solid immunity in the population and China should redesign the optimal time interval for MuCV immunization to reduce the incidence of mumps in the future.

## Data availability statement

The raw data supporting the conclusions of this article will be made available by the authors, without undue reservation.

## Author contributions

TY, YW, QZ, YS, and TC designed the research. QZ, TY, YW, BD, JH, WL, SY, and XG collected the data. BD, JH, TY, and ZZ analyzed the data. TC, TY, YW, and QZ wrote the manuscript. All authors read and approved the final manuscript.

## Funding

This study was supported by the Bill & Melinda Gates Foundation (INV-005834).

## Conflict of interest

The authors declare that the research was conducted in the absence of any commercial or financial relationships that could be construed as a potential conflict of interest.

## Publisher's note

All claims expressed in this article are solely those of the authors and do not necessarily represent those of their affiliated organizations, or those of the publisher, the editors and the reviewers. Any product that may be evaluated in this article, or claim that may be made by its manufacturer, is not guaranteed or endorsed by the publisher.

## Abbreviations

SEPIAR: Susceptible–Exposed–Pre-symptomatic–Infectious–Asymptomatic–Recovered
MMR: measles-mumps-rubella
MuCV: mumps-containing vaccines
MuV: Mumps virus
NPI: national immunization program
EPI: expanded immunization program
*R*_0_: basic reproduction number
*R*_*eff*_: effective reproduction number
*R*_*t*_: time-varying reproduction number
*R* ^2^: coefficient of determination.
