# Supplementary material for: Age-specific transmission dynamic of mumps: A long-term large-scale modeling study in Jilin Province, China
Source: Front Public Health. 2022 Nov 7;10:968702. doi: 10.3389/fpubh.2022.968702 (PMC9678053; doi:10.3389/fpubh.2022.968702)
Supplement: Supplementary file 1 [file Table_1.docx]

**Additional file 1: Derive** $\boldsymbol{R}_{\boldsymbol{eff}}$ **using the Next-Generation Method (NGM)**

Assuming the birth rate and death rate of population are balanced, we compute the effective reproduction number $R_{eff}$ using the Van den Driessche and Watmough approach in next-generation methods as follow. The time varying reproduction number $R\left( t \right)$ is then obtained by substituting real-time state function $S\left( t \right)$ in the $R_{eff}$ expression. The basic reproduction number $R_{0}$ is also obtained by substituting the disease-free equilibrium in the $R_{eff}$ expression.
**Step 1**

Assume that the population been divided into $n$ sub-groups ($n=1$ leads to expression for un-grouped model). First, we divide the $6*n$ variables (or sometimes called ’compartments’) $\left( S_{i},E_{i},P_{i},I_{i},A_{i},R_{i} \right), i=1,2,\cdots,n$ into two categories: the first $\left( E_{i},P_{i},I_{i},A_{i} \right), i=1,2,\cdots,n$ are infected compartments, and the second $\left( S_{i},R_{i} \right), i=1,2,\cdots,n$ are non-infected compartments.

## Step 2

Divide the derivatives of $\left( E_{i},P_{i},I_{i},A_{i} \right), i=1,2,\cdots,n$ into two parts: the first part $\mathcal{F}$ denotes the rate of newly infection, and the second part $\mathcal{V}$ denotes the transition inside the infected compartments:

$$\begin{matrix} \frac{d}{dt}\left[ \begin{matrix} E_{i} \\ P_{i} \\ I_{i} \\ A_{i} \end{matrix} \right]= & \left[ \begin{matrix} \sum_{j=1}^{n} \beta_{ji}S_{i}\left( I_{j}+k_{1}A_{j}+k_{2}P_{j} \right)-d_{r}E_{i}-\rho\omega_{1}E_{i}-\left( 1-\rho\right)\omega_{2}E_{i} \\ \left( 1-\rho\right)\omega_{2}E_{i}-d_{r}P_{i}-\omega_{3}P_{i} \\ \omega_{3}P_{i}-d_{r}I_{i}-\gamma_{1}I_{i} \\ \rho\omega_{1}E_{1}-d_{r}A_{i}-\gamma A_{i} \end{matrix} \right] \\ = & \left[ \begin{matrix} \sum_{j=1}^{n} \beta_{ji}S_{i}\left( I_{j}+k_{1}A_{j}+k_{2}P_{j} \right) \\ 0 \\ 0 \\ 0 \end{matrix} \right]-\left[ \begin{matrix} d_{r}E_{i}+\rho\omega_{1}E_{i}+\left( 1-\rho\right)\omega_{2}E_{i} \\ -\left( 1-\rho\right)\omega_{2}E_{i}+d_{r}P_{i}+\omega_{3}P_{i} \\ -\omega_{3}P_{i}+d_{r}I_{i}+\gamma_{1}I_{i} \\ -\rho\omega_{1}E_{1}+d_{r}A_{i}+\gamma A_{i} \end{matrix} \right] \\ \overset{def}{=} & \mathcal{F}_{i}-\mathcal{V}_{i} \end{matrix}$$

## Step 3

Taking derivatives with respect to infected variables $\left( E_{i},P_{i},I_{i},A_{i} \right), i=1,2,\cdots,n$ for vector $\mathcal{F}$ and $\mathcal{V}$, the jacobi matrices $F$ and $V$ are obtained:

$$F=\left[ \begin{matrix} F_{11} & F_{12} & \cdots& F_{1n} \\ F_{21} & F_{22} & \cdots& F_{2n} \\ \vdots& \vdots& \ddots& \vdots\\ F_{n1} & F_{n2} & \cdots& F_{nn} \end{matrix} \right], V=\left[ \begin{matrix} V_{11} & V_{12} & \cdots& V_{1n} \\ V_{21} & V_{22} & \cdots& V_{2n} \\ \vdots& \vdots& \ddots& \vdots\\ V_{n1} & V_{n2} & \cdots& V_{nn} \end{matrix} \right]$$

where

$$\begin{matrix} F_{ij}= & \left[ \begin{matrix} \partial\mathcal{F}_{i}\left( 1 \right)/\partial E_{j} & \partial\mathcal{F}_{i}\left( 1 \right)/\partial P_{j} & \partial\mathcal{F}_{i}\left( 1 \right)/\partial I_{j} & \partial\mathcal{F}_{i}\left( 1 \right)/\partial A_{j} \\ \partial\mathcal{F}_{i}\left( 2 \right)/\partial E_{j} & \partial\mathcal{F}_{i}\left( 2 \right)/\partial P_{j} & \partial\mathcal{F}_{i}\left( 2 \right)/\partial I_{j} & \partial\mathcal{F}_{i}\left( 2 \right)/\partial A_{j} \\ \partial\mathcal{F}_{i}\left( 3 \right)/\partial E_{j} & \partial\mathcal{F}_{i}\left( 3 \right)/\partial P_{j} & \partial\mathcal{F}_{i}\left( 3 \right)/\partial I_{j} & \partial\mathcal{F}_{i}\left( 3 \right)/\partial A_{j} \\ \partial\mathcal{F}_{i}\left( 4 \right)/\partial E_{j} & \partial\mathcal{F}_{i}\left( 4 \right)/\partial P_{j} & \partial\mathcal{F}_{i}\left( 4 \right)/\partial I_{j} & \partial\mathcal{F}_{i}\left( 4 \right)/\partial A_{j} \end{matrix} \right]=\left[ \begin{matrix} 0 & k_{2}\beta_{ji}S_{i} & \beta_{ji}S_{i} & k_{1}\beta_{ji}S_{i} \\ 0 & 0 & 0 & 0 \\ 0 & 0 & 0 & 0 \\ 0 & 0 & 0 & 0 \end{matrix} \right], \\ V_{ij}= & \left[ \begin{matrix} \partial\mathcal{V}_{i}\left( 1 \right)/\partial E_{j} & \partial\mathcal{V}_{i}\left( 1 \right)/\partial P_{j} & \partial\mathcal{V}_{i}\left( 1 \right)/\partial I_{j} & \partial\mathcal{V}_{i}\left( 1 \right)/\partial A_{j} \\ \partial\mathcal{V}_{i}\left( 2 \right)/\partial E_{j} & \partial\mathcal{V}_{i}\left( 2 \right)/\partial P_{j} & \partial\mathcal{V}_{i}\left( 2 \right)/\partial I_{j} & \partial\mathcal{V}_{i}\left( 2 \right)/\partial A_{j} \\ \partial\mathcal{V}_{i}\left( 3 \right)/\partial E_{j} & \partial\mathcal{V}_{i}\left( 3 \right)/\partial P_{j} & \partial\mathcal{V}_{i}\left( 3 \right)/\partial I_{j} & \partial\mathcal{V}_{i}\left( 3 \right)/\partial A_{j} \\ \partial\mathcal{V}_{i}\left( 4 \right)/\partial E_{j} & \partial\mathcal{V}_{i}\left( 4 \right)/\partial P_{j} & \partial\mathcal{V}_{i}\left( 4 \right)/\partial I_{j} & \partial\mathcal{V}_{i}\left( 4 \right)/\partial A_{j} \end{matrix} \right]=\delta_{ij}\left[ \begin{matrix} d_{r}+\rho\omega_{1}+\left( 1-\rho\right)\omega_{2} & 0 & 0 & 0 \\ -\left( 1-\rho\right)\omega_{2} & d_{r}+\omega_{3} & 0 & 0 \\ 0 & -\omega_{3} & d_{r}+\gamma_{1} & 0 \\ -\rho\omega_{1} & 0 & 0 & d_{r}+\gamma\end{matrix} \right] \end{matrix}$$

$\delta_{ij}$ is the Kronecker Delta.
The inverse of $V_{jj}$ is further computed:

$$\begin{matrix} V_{jj}^{-1}= & \left[ \begin{matrix} \frac{1}{d_{r}+\rho\omega_{1}+\left( 1-\rho\right)\omega_{2}} & 0 & 0 & 0 \\ \frac{\omega_{2} \left( 1-\rho\right)}{\left( d_{r}+\omega_{3} \right) \left( d_{r}+\omega_{1} \rho+\left( 1-\rho\right)\omega_{2} \right)} & \frac{1}{d_{r}+\omega_{3}} & 0 & 0 \\ \frac{\omega_{2} \omega_{3} \left( 1-\rho\right)}{\left( d_{r}+\gamma_{1} \right) \left( d_{r}+\omega_{3} \right) \left( d_{r}+\omega_{1} \rho+\left( 1-\rho\right)\omega_{2} \right)} & \frac{\omega_{3}}{\left( d_{r}+\gamma_{1} \right) \left( d_{r}+\omega_{3} \right)} & \frac{1}{d_{r}+\gamma_{1}} & 0 \\ \frac{\omega_{1} \rho}{\left( d_{r}+\gamma\right) \left( d_{r}+\omega_{1} \rho+\left( 1-\rho\right)\omega_{2} \right)} & 0 & 0 & \frac{1}{d_{r}+\gamma} \end{matrix} \right] \end{matrix}$$

## Step 4

Construct the next generation matrix $M=FV^{-1}$.
Block matrices $M$, $F$ and $V$:

$$M=\left[ \begin{matrix} M_{11} & M_{12} & \cdots& M_{1n} \\ M_{21} & M_{22} & \cdots& M_{2n} \\ \vdots& \vdots& \ddots& \vdots\\ M_{n1} & M_{n2} & \cdots& M_{nn} \end{matrix} \right]=\left[ \begin{matrix} F_{11} & F_{12} & \cdots& F_{1n} \\ F_{21} & F_{22} & \cdots& F_{2n} \\ \vdots& \vdots& \ddots& \vdots\\ F_{n1} & F_{n2} & \cdots& F_{nn} \end{matrix} \right]\left[ \begin{matrix} V_{11}^{-1} & & & \\ & V_{22}^{-1} & & \\ & & \ddots& \\ & & & V_{nn}^{-1} \end{matrix} \right],$$

where

$$\begin{matrix} M_{ij} & =\sum_{k=1}^{n} F_{ik}V_{kj}^{-1}=F_{ij}V_{jj}^{-1}=\left[ \begin{matrix} a_{ij} & b_{ij} & c_{ij} & d_{ij} \\ 0 & 0 & 0 & 0 \\ 0 & 0 & 0 & 0 \\ 0 & 0 & 0 & 0 \end{matrix} \right], \end{matrix}$$

$$\begin{matrix} a_{ij}= & \frac{\omega_{2} \left( 1-\rho\right)k_{2}\beta_{ji}S_{i}}{\left( d_{r}+\omega_{3} \right) \left( d_{r}+\omega_{1} \rho+\left( 1-\rho\right)\omega_{2} \right)}+\frac{\omega_{2} \omega_{3} \left( 1-\rho\right)\beta_{ji}S_{i}}{\left( d_{r}+\gamma_{1} \right) \left( d_{r}+\omega_{3} \right) \left( d_{r}+\omega_{1} \rho+\left( 1-\rho\right)\omega_{2} \right)}+\frac{\omega_{1} \rho k_{1}\beta_{ji}S_{i}}{\left( d_{r}+\gamma\right) \left( d_{r}+\omega_{1} \rho+\left( 1-\rho\right)\omega_{2} \right)}, \\ b_{ij}= & \frac{k_{2}\beta_{ji}S_{i}}{d_{r}+\omega_{3}}+\frac{\omega_{3}\beta_{ji}S_{i}}{\left( d_{r}+\gamma_{1} \right)\left( d_{r}+\omega_{3} \right)}, \\ c_{ij}= & \frac{\beta_{ji}S_{i}}{d_{r}+\gamma_{1}}, \\ d_{ij}= & \frac{k_{1}\beta_{ji}S_{i}}{d_{r}+\gamma}. \end{matrix}$$

## Step 5

Compute the eigenvalues:

$$R_{eff}=\lambda_{max}\left( M \right)$$

In most cases, the leading eigenvalue cannot be formulated as eigenvalues of sub-matrices $M_{ij}$, however, there are circumstances that $\lambda_{max}\left( M \right)$still have a simple formulation.
Noticed that $V_{jj}=V_{11}$ for all $j$, i.e. group irrelevant, let $B$ be a matrix of entries $b_{ij}=\beta_{ji}S_{i}$; let matrix $C=M_{ij}/\left( \beta_{ji}S_{i} \right)$ is a group irrelevant matrix, then :

$$M=B\otimes C,$$

where $\otimes$ is the Kronecker product.

Using properties of Kronecker product (<http://matrixcookbook.com>), one obtains:

$$\begin{matrix} R_{eff} & =\lambda_{\text{max}}\left( M \right) \\ & =\lambda_{\text{max}}\left( B \right)\lambda_{\text{max}}\left( C \right) \\ & =\lambda_{\text{max}}\left( B \right)\left[ \frac{\omega_{2} \left( 1-\rho\right)k_{2}}{\left( d_{r}+\omega_{3} \right) \left( d_{r}+\omega_{1} \rho+\left( 1-\rho\right)\omega_{2} \right)}+\frac{\omega_{2} \omega_{3} \left( 1-\rho\right)}{\left( d_{r}+\gamma_{1} \right) \left( d_{r}+\omega_{3} \right) \left( d_{r}+\omega_{1} \rho+\left( 1-\rho\right)\omega_{2} \right)}+\frac{\omega_{1} \rho k_{1}}{\left( d_{r}+\gamma\right) \left( d_{r}+\omega_{1} \rho+\left( 1-\rho\right)\omega_{2} \right)} \right] \end{matrix}$$

Let $R$ be a matrix with entries:

$$\begin{matrix} R_{ij}= & \lambda_{max}\left( M_{ij} \right) \\ = & \frac{\omega_{2} \left( 1-\rho\right)k_{2}\beta_{ji}S_{i}}{\left( d_{r}+\omega_{3} \right) \left( d_{r}+\omega_{1} \rho+\left( 1-\rho\right)\omega_{2} \right)}+\frac{\omega_{2} \omega_{3} \left( 1-\rho\right)\beta_{ji}S_{i}}{\left( d_{r}+\gamma_{1} \right) \left( d_{r}+\omega_{3} \right) \left( d_{r}+\omega_{1} \rho+\left( 1-\rho\right)\omega_{2} \right)}+\frac{\omega_{1} \rho k_{1}\beta_{ji}S_{i}}{\left( d_{r}+\gamma\right) \left( d_{r}+\omega_{1} \rho+\left( 1-\rho\right)\omega_{2} \right)}, \end{matrix}$$

then, we can see that $R_{eff}=\lambda_{max}\left( R \right)$.

## Step 6

There are three kinds of reproduction numbers: the basic reproduction number $R_{0}$, the effective reproduction number $R_{eff}$, and the time-varying or real-time reproduction number $R\left( t \right)$. Both $R_{0}$ and $R_{eff}$ are functions of status (population status of entirely susceptible, non-intervention and social status of the certain economy and contact pattern for $R_{0}$; specific status for $R_{eff}$), while $R\left( t \right)$ is a function of time.
Once the status ($S\left( t \right)$, $\beta\left( t \right)$,...) is expressed as functions of time (e.g. via numerical solution of an ordinary differential equation), then we can immediately get $R\left( t \right)$ from $R_{eff}$ by substitution. If the status at time 0 is simplified as entirely susceptible, non-intervention, then $R_{0}$ could be obtained by letting $t=0$ in the expression of $R\left( t \right)$
